# Reducing the Use of Physical Restraints in Patients With Dementia Who Are Admitted to Acute Old Age Psychiatry Wards With Agitation

**DOI:** 10.1192/bjo.2024.374

**Published:** 2024-08-01

**Authors:** Richard Goveas, Damien Lai, Fengyuan Yao

**Affiliations:** Institute of Mental Health, Singapore

## Abstract

**Aims:**

The Institute of Mental Health is the only tertiary Psychiatric Hospital in Singapore. It has two 29 bedded inpatient wards which provide acute care for the elderly with severe mental health conditions including dementia. Restraints are one of the methods employed in managing agitation in patients with dementia. The physical consequences of restraints are reduced mobility resulting in decreased muscle tone and mass, bone demineralisation, orthostatic hypotension, and atelectasis. This results in patients who are more prone to falls, aspiration pneumonia, deep vein thrombosis/pulmonary embolism and ulcers. The psychological consequences include aggravating agitation, feelings of humiliation, negative emotions like anger and despair. Hence, we embarked on a program to reduce the use of physical restraints in the management of agitation in patients with dementia.

**Methods:**

Baseline restraint hours were collected from 7am to 9pm for all dementia patients who were restrained for agitation for a period of 5 months. Patients on Geri chair with seatbelt used primarily for fall prevention were not included. The Pittsburgh Agitation Scale was used to measure agitation.

The nursing staff were trained on the Enriched model for targeting behaviour and on the VIPS (Valuing people, Individualised care, Personal perspectives, Social environment) framework for person centred care. Restraint hours were collected post intervention as well as benzodiazepine usage data over both periods to monitor any changes in the usage.

**Results:**

The baseline data (preintervention) over a 5-month period determined that patients with dementia who were agitated were being restrained on an average for 3.33 hrs per day from the period of January to May 2021. Following the training of nursing staff on the enriched model of care and the use of VIPS framework for person centered care the restraint hours reduced to 1.48hr per day over 5 months from January to May 2022. Benzodiazepines usage went down from 0.34mg at baseline to 0.17mg per dementia patient per day post intervention.

**Conclusion:**

Nonpharmacological interventions (enriched model and VIPS framework for person centered care) using a multidisciplinary team approach is effective in the management of agitation resulting from dementia and should be used as a first line in the management of such conditions.

